# PfSMAD4 plays a role in biomineralization and can transduce bone morphogenetic protein-2 signals in the pearl oyster *Pinctada fucata*

**DOI:** 10.1186/s12861-016-0110-4

**Published:** 2016-04-26

**Authors:** Mi Zhao, Yu Shi, Maoxian He, Xiande Huang, Qi Wang

**Affiliations:** CAS Key Laboratory of Tropical Marine Bio-resources and Ecology, South China Sea Institute of Oceanology, Chinese Academy of Sciences, Guangzhou, 510301 China; University of Chinese Academy of Sciences, Beijing, 100049 China

**Keywords:** SMAD4, Biomineralization, BMP signaling pathway, *Pinctada fucata*

## Abstract

**Background:**

Mollusca is the second largest phylum in nature. The shell of molluscs is a remarkable example of a natural composite biomaterial. Biomineralization and how it affects mollusks is a popular research topic. The BMP-2 signaling pathway plays a canonical role in biomineralization. SMAD4 is an intracellular transmitter in the BMP signaling pathway in mammals, and some genomic data show SMAD4’s involvment in BMP signaling in invertbrates, but whether SMAD4 plays a conservative role in pearl oyster, *Pinctada fucata*, still need to be tested.

**Results:**

In this study, we identified a *SMAD4* gene (hereafter designated *PfSMAD4*) in pearl oyster *Pinctada fucata.* Bioinformatics analysis of *PfSMAD4* showed high identity with its orthologs. PfSMAD4 was located in the cytoplasm in immunofluorescence assays and analyses of *PfSMAD4* mRNA in tissues and developmental stages showed high expression in ovaries and D-shaped larvae. An RNA interference experiment, performed by *PfSMAD4* double-stranded RNA (dsRNA) injection, demonstrated inhibition not only of nacre growth but also organic sheet formation with a decrease in *PfSMAD4* expression. A knockdown experiment using *PfBMP2* dsRNA showed decreased *PfBMP2* and *PfSMAD4* mRNA and irregular crystallization of the nacreous layer using scanning electron microscopy. In co-transfection experiments, PfBMP2-transactivated reporter constructs contained *PfSMAD4* promoter sequences.

**Conclusions:**

Our results suggest that PfSMAD4 plays a role in biomineralization and can transduce BMP signals in *P. fucata.* Our data provides important clues about the molecular mechanisms that regulate biomineralization in pearl oyster.

## Background

Pearl oyster, *Pinctada fucata,* is distributed over the southern coast of China and is the most popular farming shellfish for pearl production. The plain outer surface of pearl oyster shells conceal the lustrous beauty of the mother-of-pearl lining ‘nacre’. It combines a high mechanical strength similar to many ceramics, with elasticity, reducing the brittleness of the shell [1, 2]. The nacreous layer of molluskan shells, which consist of highly oriented aragonitic crystals and an organic matrix (including chitin and proteins), is a product of biomineralization [3–5].

Bone morphogenic proteins (BMP) are the largest subgroup in the transforming growth factor-beta (TGF-β) superfamily [6] and play a canonical role in biomineralization [7, 8]. In the BMP family, BMP-2 has one of the strongest signals for stimulating biomineralization. BMP-2 stimulates bone or tooth mineralization via the canonical BMP pathway [9–11]; SMAD 1, 5, and presumably 8, propagate BMP signals and are structurally related to Mad that acts downstream of Dpp, a BMP homolog in *Drosophila* [12]. SMAD4 is the only Co-SMAD in mammals [13], and Medea acts as a common SMAD in flies [14]. In the cytoplasm, receptor-regulated SMADs (R-SMADs) are directly phosphorylated by BMP-like ligands and then associate with common SMADs (Co-SMADs) that are essential to distinct signaling pathways. The heteromeric complexes are translocated to the nucleus, where they regulate transcription of target genes in concert with other transcription factors [15, 16].

SMADs have a domain structure consisting of highly conserved amino (NH_2_)- and (COOH)-terminal regions, referred to as Mad homology 1 (MH1) and MH2 domains [17, 18], respectively. The MH1 domain can bind to specific DNA sequences in the nucleus and the MH2 domain is responsible for interaction with other SMAD proteins [19].

Accumulating examples show that BMP orthologs play important roles in biomineralization in mollusca [20–25]. In previous studies, the *BMP-2* gene of *P. fucata* has been identified and defined as *PfBMP2* [26]. Further studies showed that a purified recombinant 10-kD mature fragment of PfBMP2 could induce osteogenic differentiation in C3H10T1/2 [27], demonstrating that PfBMP2 is conserved in terms of its function in the formation of hard tissuePreliminary studies of SMAD4 genes in *Crassostrea gigas* and *Lingula anatina* show their potential involvement in shell formation [28, 29], and Luo *et al.* showed SMAD4’s involvment in BMP-2 signaling based on Mollusca and brachiopod genomes [29]. Although a SMAD4 homolog was found in *P. fucata* (designated PfSMAD4), whether the SMAD4 protein has the same function as their homologs still needs to be tested. In this study, we investigated if PfSMAD4 played a role in biomineralization. Additionally, we identified that PfBMP2 could activate the promoter of PfSMAD4, and *PfSMAD4* expression decreased after interfering with the expression of *PfBMP2*.

## Results

### Sequence analysis of PfSMAD4

**Fig. 1:**
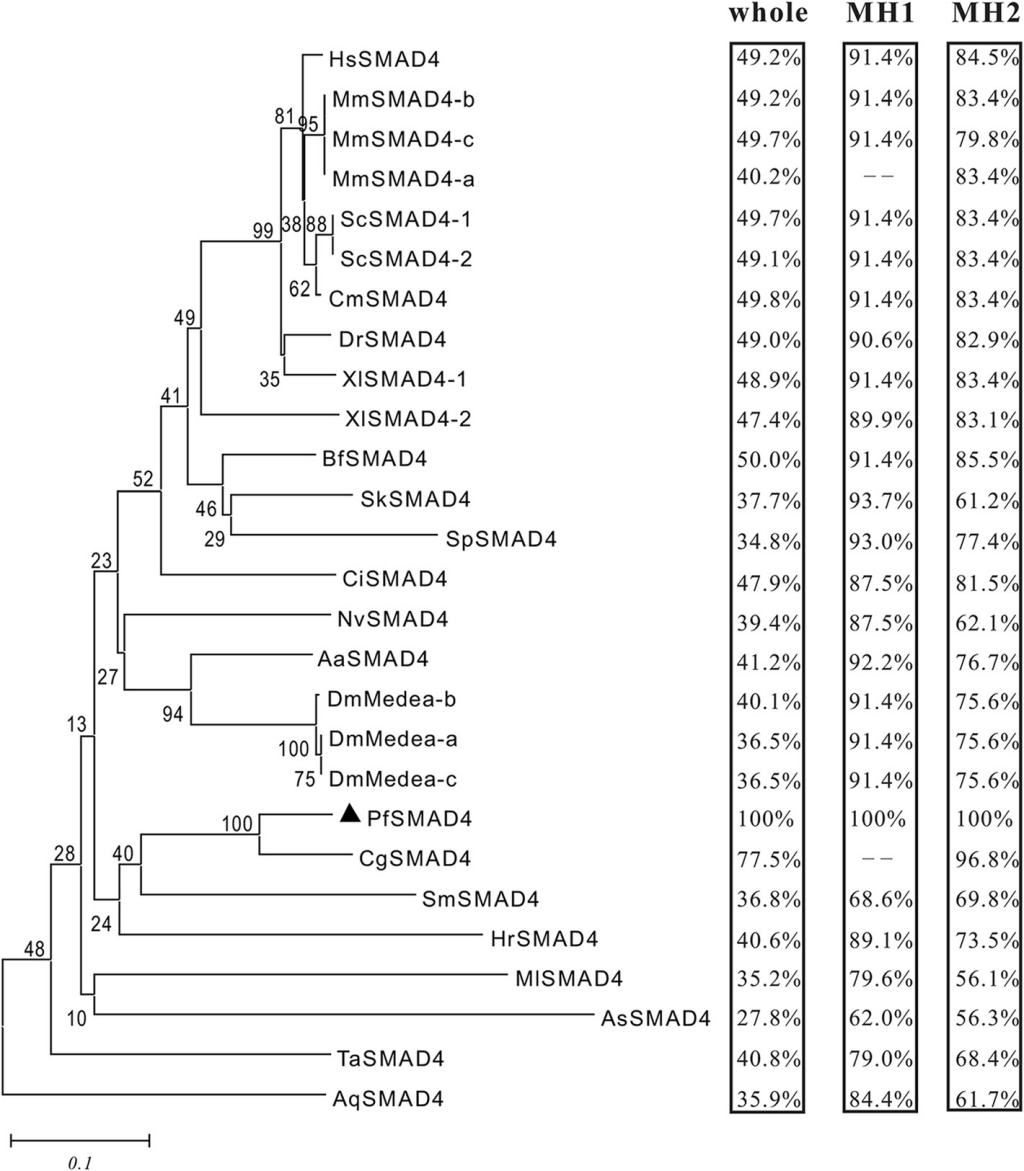
Bioinformatics analysis of PfSMAD4. Phylogenetic analysis of the SMAD4 family. The phylogenetic tree was constructed by MEGA 5.0 using the neighbor-joining method with 1000 bootstrap replicates. The number shown at each branch indicates the bootstrap value (%). Percentages refer to identity values. The left frame showed the identities of the whole PfSMAD4 sequence with its orthologs. The middle frame: MH1 domain; the right frame: MH2 domain. These SMAD4 amino acid sequences using in the alignment and phylogenetic analysis are from HsSMAD4 (*Homo sapiens*, AAH02379.1), MmSMAD4-a (*Mus musculus*, EDL09559.1), MmSMAD4-b (*Mus musculus*, EDL09560.1), MmSMAD4-c (*Mus musculus*, EDL09561.1), ScSMAD4-1 (*Serinus canaria*, XP_009098382.1), ScSMAD4-2 (*Serinus canaria*, XP_009098384.1), CmSMAD4-a (*Chelonia mydas*, XP_007063905.1), DrSMAD4-a (*Danio rerio*, NP_001116172.1), XlSMAD4-1 (*Xenopus laevis*, NP_001090536.1), XlSMAD4-2 (*Xenopus laevis*, NP_001084387.1), BfSMAD4 (*Branchiostoma floridae*, AEU03847.1), SkSMAD4 (*Saccoglossus kowalevskii,* XP_002740765.2), SpSMAD4 (*Strongylocentrotus purpuratus,* XP_780740.3), CiSMAD4 (*Ciona intestinalis*, NP_001071944.1), NvSMAD4 (*Nematostella vectensis,* EDO31382.1), AaSMAD1/5 (*Aedes aegypti,* XP_001664103.1), DmMedea-a (*Drosophila melanogaster,* AAF57172.1), DmMedea-b (*Drosophila melanogaster,* AAN14277.1), DmMedea-c (*Drosophila melanogaster,* AAN14278.2), CgSMAD4 (*Crassostrea gigas*, EKC24133.1), PfSMAD4 (*Pinctada fucata*, AGY49100.1), SmSMAD4 (*Schistosoma mansoni,* XP_002574840.1), HrSMAD4 (*Helobdella robusta,* ESN93792.1), MlSMAD1/5 (*Mnemiopsis leidyi,* AEP16392.1), AsSMAD4 (*Ascaris suum,* ERG79533.1), TaSMAD4 (*Trichoplax adhaerens,* XP_002116214.1) and AqSMAD4 (*Amphimedon queenslandica*, XP_003388571.1)

Phylogenetic analysis showed that the PfSMAD4 sequence was most closely related to that of *Crassostrea gigas*, which also belongs to bivalves. The relationships displayed in the phylogenic tree are generally in agreement with those of traditional taxonomy. Homology analysis revealed that the whole PfSMAD4 sequence shared 27.8–77.5 % identity to other known SMAD4 sequences, while the MH1 domain shared 62–93.7 % identity and MH2 domain shared 56.1–96.8 % (Fig. 1).

### *PfSMAD4* expression in tissues and developmental stages

**Fig. 2:**
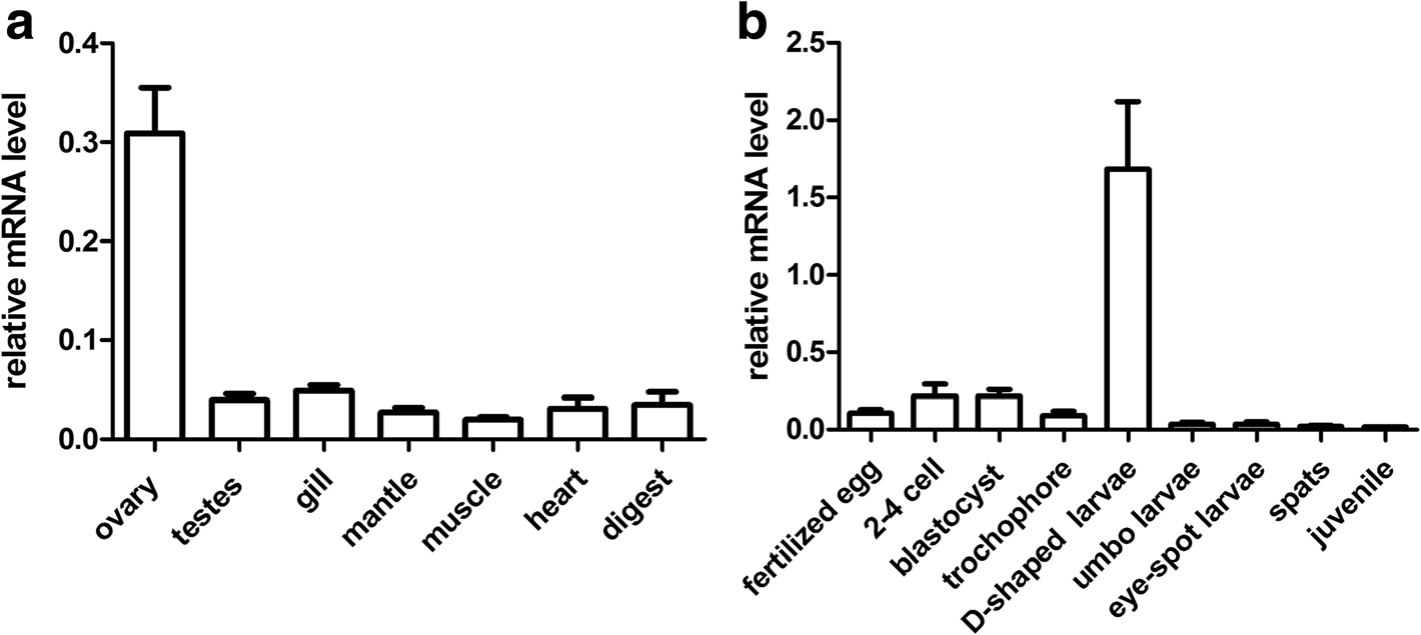
Expression of *PfSMAD4* mRNA in various tissues (**a**) and at the developmental stages of *Pinctada fucata* (**b**). The mRNA levels were quantified by qPCR. The results are expressed as fold-change. Each bar represents the mean ± S.E.M (*n* = 3)

To investigate the expression pattern of *PfSMAD4* among various tissues and developmental stages in pearl oyster, qPCR analysis was performed with gene specific primers. The expression of *PfSMAD4* was abundant in all tissues examined, including ovary, testis, gill, mantle, heart, and digestive. *PfSMAD4* was expressed at particularly high levels in ovaries (Fig. 2a). High expression levels were also observed in all developmental stages investigated, particularly in the D-shaped larvae (Fig. 2b).

### PfSMAD4 is localized to the cytoplasm

**Fig. 3:**
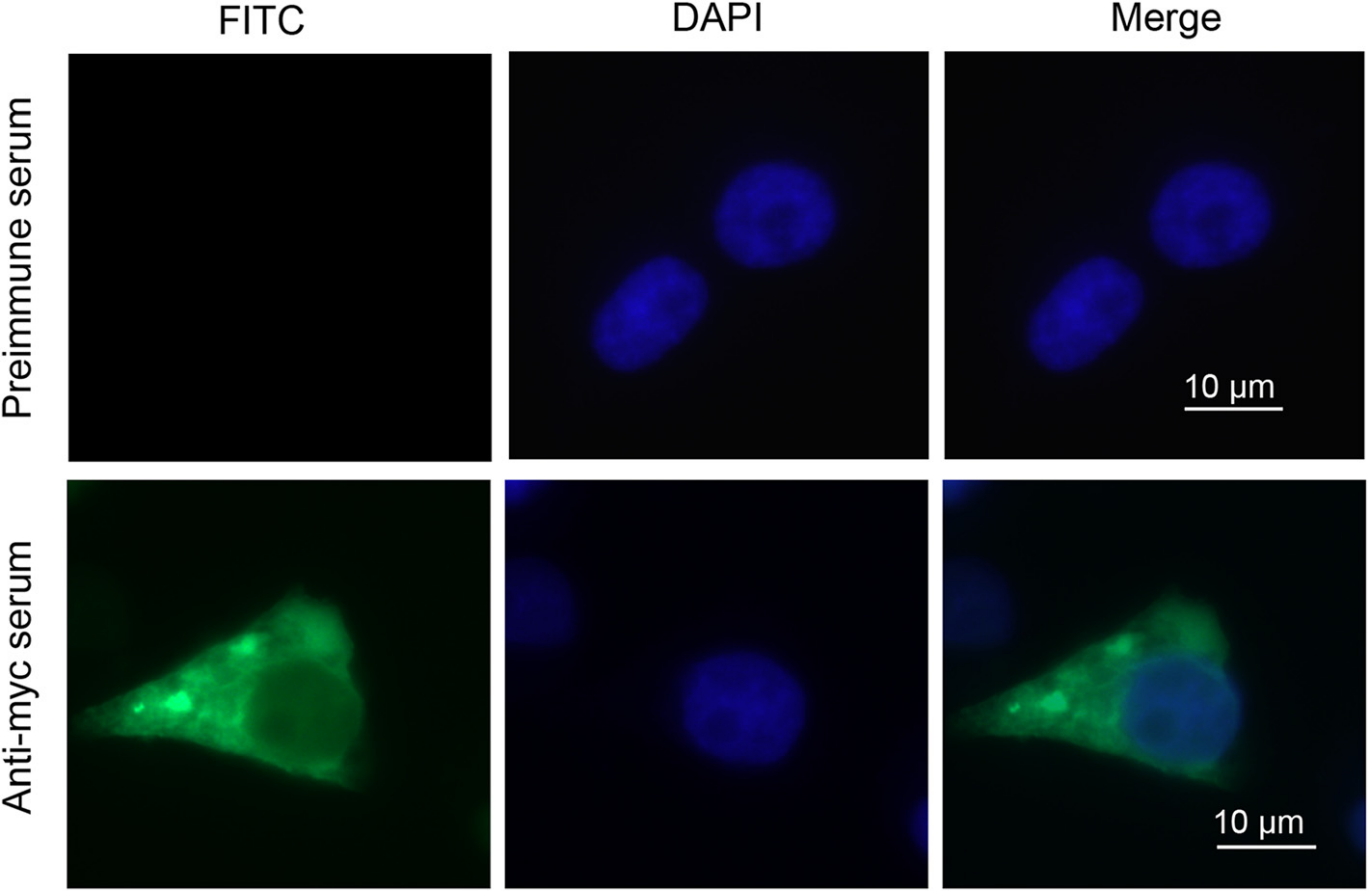
The sub-cellular localization of PfSMAD4 in HEK293T cells. Indirect immunofluorescence staining of PfSMAD4 using mouse anti-myc antibody and FITC-conjugated goat anti-mouse antibodies. Preimmune mice serum was used as control (upper row), and blue images show the location of the nucleus stained by DAPI

Subcellular localization of PfSMAD4 was investigated by immunofluorescence assays. The results indicated that PfSMAD4 was located in the cytoplasm (Fig. 3 lower row). No fluorescence signal was detected in the control cells detected by the preimmune mouse serum (Fig. 3, upper row). In an uninduced state, the SMADs are retained in the cytoplasm [30–32]. The immunofluorescence assays showed PfSMAD4 was seen in the cytoplasm of the cells; this tallied with the views above.

### Knockdown of *PfSMAD4* leads to disorder of the nacreous layer

**Fig. 4:**
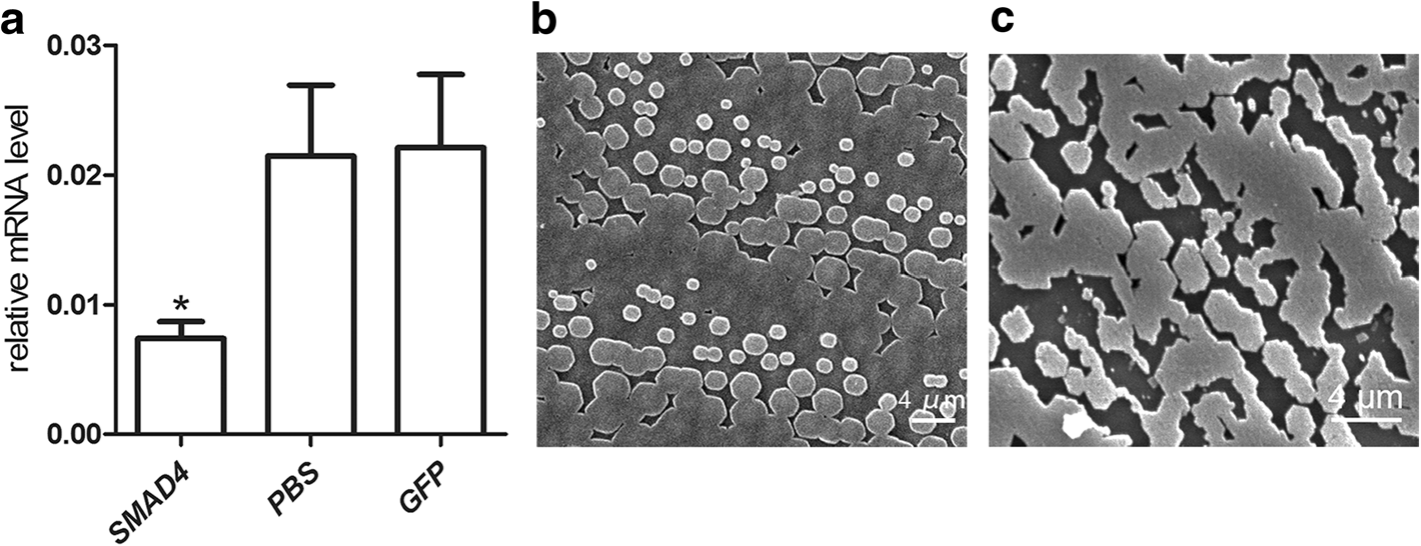
Knockdown of the *PfSMAD4* gene by means of RNAi. **a** The expression levels of *PfSMAD4* mRNA in the mantle were determined with qPCR 7 days after injection. Five oysters were used in each experiment. Statistically significant differences were analyzed by means of one-way analysis of variance. Asterisk indicates a significant reduction (*P* < 0.05) as compared with PBS-injected oysters. **b** and **c** SEM images of the surface of the nacreous layer of the oysters injected with PBS and 80 μg of *PfSMAD4* dsRNA respectively

We tested the function of PfSMAD4 in biomineralization using RNAi technology. The controls were PBS and dsRNA-GFP; GFP was not expressed in *P. fucata*. The *PfSMAD4* dsRNA was injected into *P. fucata*, and qPCR was used to measure expression levels of the *PfSMAD4* gene 7 days after dsRNA injection. The *PfSMAD4* expression levels in the PfSMAD4-dsRNA injected group were suppressed by approximately 70 %, compared with the PBS group (Fig. 4a). We also observed the inner surface structure of the shells. The surfaces of the shells in the control groups (PBS and dsRNA-GFP) had a normal well-defined microstructure (Fig. 4b). The shell surface in *PfSMAD4* dsRNA injected groups, stopped regular crystallization and formed a mass without clear boundaries (Fig. 4c).

### Knockdown of *PfBMP2* leads to reduced *PfSMAD4* expression

**Fig. 5:**
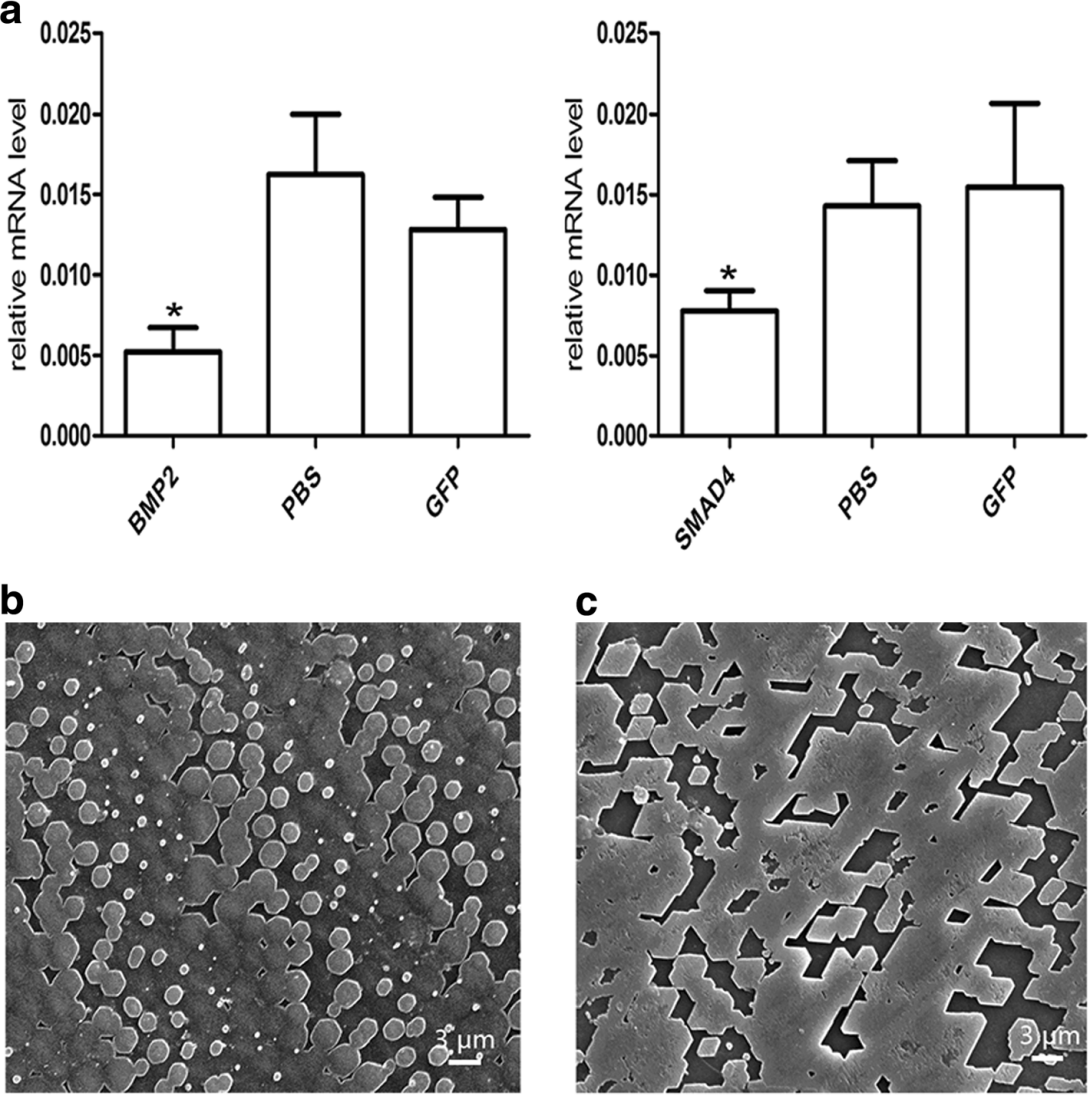
Knockdown of the *PfBMP2* gene by means of RNAi. **a** The expression levels of *PfBMP2* and *PfSMAD4* mRNA in the mantle were determined with qPCR 7 days after injection. Five oysters were used in each experiment. Statistically significant differences were analyzed by means of one-way analysis of variance. Asterisk indicates a significant reduction (*P* < 0.05) as compared with PBS-injected oysters. **b** and **c** SEM images of the surface of the nacreous layer of the oysters injected with PBS and 80 μg of *PfBMP* dsRNA × 1000 magnification, respectively

We then tested whether PfSMAD4 transduces PfBMP2 signals using RNAi technology on the *PfBMP2* gene. The *PfBMP2* dsRNA was injected into the muscle of *P. fucata*, and qPCR was used to measure expression levels of the *PfBMP2* and *PfSMAD4* genes. *PfBMP2* and *PfSMAD4* expression levels of the 80 μg-dsRNA injected groups were suppressed by approximately 70 % and 50 %, respectively, compared with the PBS or dsRNA-GFP injected groups (Fig. 5a). Incidentally, we also observed the inner surface structure of the shells after dsRNA injection using SEM. The surfaces of shells in the control groups (PBS and dsRNA-GFP) were normal (Fig. 5b). In the *PfBMP2* dsRNA injected groups, the growth of the nacre tablets was disrupted (Fig. 5c), resembling the nacre pattern after *PfSMAD4* interference. These results further reinforce the concept that BMP2 has a function in pearl oyster biomineralization. On the other hand, this tight correlation between the expression of *PfBMP2* and *PfSMAD4* at the molecular level, and a similar pattern after knockdown, strongly suggested that PfBMP2 was the upstream regulation gene of PfSMAD4.

### PfBMP2 activates PfSMAD4-specific reporter genes

**Fig. 6:**
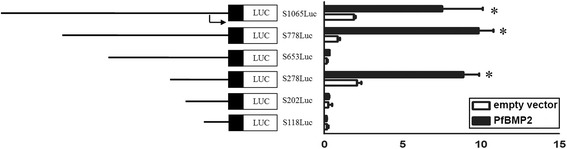
PfBMP2 activates *PfSMAD4* promoter in HEK293T cells. Left graph: indicated segments from the 5'-flanking region of the *PfSMAD4* gene linked to pGL3 basic encoding luciferase. Right graph: the synthetic PfSMAD4-Luc reporter was transfected into HEK293T cells in the absence (vector) or presence of expression vectors for *PfBMP2*. Forty-eight hours after transfection, whole cell lysates were prepared and analyzed for luciferase activity. The bars indicate relative luciferase activity. Normalized luciferase activities are shown as the mean ± S.E.M (*n* = 3). Statistically significant differences were analyzed by means of the Student’s t-test. Asterisk indicates a significant reduction (*P* < 0.05)

A series of 5'-deletion mutants were prepared to determine whether the *PfSMAD4* promoter might harbor cis-regulatory DNA sequences critical for transactivation by PfBMP2 (Fig. 6a, left graph). Each deletion mutant was co-transfected into HEK293T cells along with either pCDNA3.1-BMP2 or pCDNA3.1.

S278Luc is the basic promoter of the *PfSMAD4* promoter. Deletions of the region from −778 to −653 resulted in 40-fold increases in promoter activity, suggesting that these regions function as silencers in controlling *PfSMAD4* gene transactivation (Fig. 6, right graph). Over-expression of pCDNA3.1 vector had no obvious effect on the activities of S278Luc, S778Luc and S1065Luc, but when transfected with pCDNA3.1-BMP2, their activity significantly increased (Fig. 6, right graph). The results presented in this report show that PfBMP2, when expressed in transiently transfected mammalian cells, can activate transcription from the *PfSMAD4* promoter and cis-regulatory DNA sequences may exist in the region from −202 to −278.

## Discussion

### PfSMAD4 plays a role in biomineralization

The *PfSMAD4* gene shows high expression in mantle and D-shaped larvae stages. The mantle tissue stage corresponds to shell formation and the D-shaped larval stage is a period in which mineral materials largely accumulate. These results may suggest that *PfSMAD4* exerts a function in shell formation not only in the adult but also during the embryonic stage. High expression level of the SMAD4 gene reported in the shell fields of embryos at different stages in *Crassostrea gigas* [29] is consistent with our study. The high expression in the ovary may indicate that PfSMAD4 functions in reproduction and development.

It is well known that TGF-β/BMP signaling play important roles in osteoblast differentiation and bone formation [33]. As a common mediator Smad of TGF-β and BMP signaling, SMAD4 is also required for maintaining normal bone homeostasis. Conditional deletion of Smad4 in osteoblasts leads to lower bone mineral density, decreased bone volume, decreased bone formation rate, and a reduced number of osteoblasts [34]. Mutations at a single codon in Mad homology 2 domain of SMAD4 can cause Myhre syndrome, which is a developmental disorder characterized by a shortness in stature, hands, feet, and so on [35]. Interference of *PfSMAD4* caused nacre disorder showed that PfSMAD4 played a role in biomineralization in *P. fucata.*

### Conserved BMP2/SMAD4 signaling pathway in *P. fucata*

In recent years, many alternatively spliced SMAD4 variants have been found in many species [36–39]. Most isoforms lack one or more in-frame exons, compared with the full-length transcripts, and the activities of their encoded proteins depends on which region of the SMAD protein is missing or affected [40]. Comparison of the deduced amino acid sequence of PfSMAD4 with SMAD4 from other organisms showed that PfSMAD4 has an overall 27.8–77.5 % identity with known sequences. The MH1 domain and MH2 domain showed higher identities, ranging from 62 to 93.7 % and 56.1–96.8 %, respectively. The high identities of the MH1 and MH2 domains of SMADs imply a highly conserved structure, further suggesting a conservation in function. The SMAD4 sequence is conserved in eukaryotes from sponges to mammals and the PfSMAD4 has a high similarity to vertebrate SMAD4, confirming the hypothesis by Westbroek et al. [41] that human and pearl oyster may have homogeneous signal transmitters in biomineralization.

Many developmental mechanisms have shown to be conserved throughout evolution [42]. Gabrielle et al. [43] demonstrated that the BMP signaling pathway was in place prior to the divergence in the line of Cnidaria to the higher Metazoa, and that it has been substantially conservative during evolution. Based on Mollusca and brachiopod genomes, BMP-SMAD signaling pathway showed its conservation in verterbrates [29]. The conserved SMAD4 was identified in many invertebrates like fly [44], ascidian [45] and amphioxus [46], demonstrating a conserved function in the BMP signalling pathway. RNAi technology has been applied in investigating the function of specific genes [47] and it has been used successfully in Mollusca [48–51]. As a potential signal transducing molecule, SMAD4 protein is expected to be co-expressed with the BMP signaling molecule. The interference of *PfBMP2* mRNA led to reduced *PfSMAD4* expression, indicating that *PfSMAD4* could transduce a BMP2 signaling pathway. Moreover, the nacre pattern after *PfSMAD4* interference bore similar resemblance to that after *PfBMP2* interference, highlighting an essential role of PfSMAD4 in mediating the BMP signaling pathway in *P. fucata.* These results are reinforced by our luciferase assays showing PfBMP2 could activate the *PfSMAD4* promoter.

## Conclusions

Our results suggest that PfSMAD4 plays a role in biomineralization and can transduce BMP signals in *P. fucata.* Our data provide important clues about the molecular mechanisms that regulate biomineralization in pearl oyster.

## Methods

### Bioinformatics analysis of PfSMAD4

PfSMAD4 sequence was obtained from GenBank, accession number AGY49100.1. Multiple sequence alignments of the deduced amino acids were performed using ClustalX2 [52] and protein domains were predicted by ExPASy translate tool (http://web.expasy.org/translate/). A neighbor-joining phylogenetic tree was constructed using the MEGA5.0 package [53]. Reliability of branching was tested using bootstrap re-sampling with 1000 pseudo-replicates.

### Cloning the 5' flanking region of the *PfSMAD4* gene

GenomeWalker libraries were constructed using a GenomeWalker Universal kit according to manufacturer’s instructions (Clontech, Mountain View, CA, USA). Pearl oyster genomic DNA (2.5–5 μg) in each reaction was digested at 37 °C overnight with a restriction enzyme. Four enzymes (*Dra*I, *Eco*RV, *Pvu*II and *Stu*I) were used in four reactions, respectively. After purification with phenol and chloroform extraction and ethanol precipitation, the digested DNA was ligated to GenomeWalker adapters (5'-GTAATACGACTCACTATAGGGCACGCGTGGTCGACGGCCCGGGCTGGT-3') at 16 °C overnight. Primers for PCR-based DNA walking in GenomeWalker libraries were gene-specific: *PfSMAD4*-specific primer 1 (5'-ACCTGCCATCCAGAGTTCTT-3') and nested primer 2 (5'-CCAGACTTTCTATGGCTCGT-3'). The longest fragment from the four genomic libraries was gel-purified, and subcloned for sequencing. According to the sequence, the nested primer 3 (5'-GGAGGTCAATTCTCGGAAAC-3') was designed. The second round PCR used nested primer 2 and nested primer 3. From two rounds of PCR, we got a 2524 bp 5' UTR-intron and a 1065 bp 5' flanking sequence [GenBank:KJ530991].

### RNA isolation and quantitative PCR analysis

*P. fucata* samples were isolated using TRIzol (Invitrogen, Carlsbad, CA, USA). Total RNA (1 μg) was treated with DNase I (Fermentas, Shenzhen, China) to prevent DNA contamination and subsequently reverse transcribed with Toyobo RT-PCR kit (Toyobo, Osaka, Japan). Quantitative PCR (qPCR) primers for tissue and developmental stage distribution were as follows: *PfSAMD4*, 5'- ATGCACCCGGTAGCTCTA-3' and 5'-TCACCGACTCCGAAACAGG-3'; *β-actin*, 5'- TGGTATGGGACAGAAGGAC-3' and 5'- GACAATGCCGTGCTCAAT -3'.

qPCR was carried out using a LightCycler 480 Real-Time PCR System (Roche, Basel, Switzerland), with SYBR green fluorescent dye, according to the manufacturer’s protocol (Toyobo). qPCR conditions were as follows: denaturation at 94 °C for 1 min, followed by 40 cycles at 94 °C for 15 s, 55 °C for 15 s and 72 °C for 60 s. We analyzed the relative gene expression using the typical cycle threshold (Ct) method (2-^ΔΔCt^ method).

### Plasmid construction

The cDNA encoding the full-length *PfBMP2* was amplified with sequence specific primers, 5'-CGGGGTACCATGATTTACGGATTTGGACAT-3' containing a *Kpn*I restriction site, and 5' -CCGCTCGAGCCGACATCCGCATCCTTC-3' containing an *Xho*I restriction site. After double digestion with *Kpn*I and *Xho*I, the cDNA was cloned in-frame into the *Kpn*I/*Xho*I sites of pcDNA3.1/myc-His (A) vector (Invitrogen). The construct was verified by sequencing. The pCDNA3.1-PfSMAD4 was constructed using the same strategy as above. Specific primers for *PfSMAD4*: F, 5'- CGGGGTACCATGACGACACAAGCACCAACG-3' (*Kpn*I restriction site is underscored) and R, 5'-CCGCTCGAGGCCTAGGAAGAATCCTCT-3’ (*Xho*I restriction site is underscored).

A 1065 bp *PfSMAD4* promoter fragment was subcloned into the *Kpn*I and *Bgl*II sites of the pGL3-basic luciferase reporter vector (Promega, Madison, WI, USA) to generate S1065Luc. The fragments of the *PfSMAD4* gene between S778Luc, S563Luc, S278Luc, S202Luc and S118Luc were amplified by PCR using S1065Luc as a template (transcriptional initiation site was defined as +1).

### Cell culture, transfection

The 293 T human kidney cell line (HEK293T) was cultured at 37 °C in a humidified atmosphere of 5 % CO_2_ using DMEM (Gibco, Grand Island, NY, USA) supplemented with 10 % FBS (Gibco), 100 IU/ml penicillin and 100 μg/ml streptomycin (Gibco). The cultures were split every 2 to 3 days. Lipofectamine 2000 (Invitrogen) was used for the DNA transfections according to the manufacturer’s protocol.

### *PfSMAD4* distribution in *P. fucata*

Adult pearl oysters (shell length 4.5–5.5 cm) were obtained from Daya Bay (China Marine Biology Research Station, South China Sea Institute of Oceanology, the Chinese Academy of Sciences) in Shenzhen, China. They were acclimated in indoor cement ponds, at ambient seawater temperature for 1 week, before the experiment. Tissue expression profiles of *PfSMAD4* were analyzed in ovaries, testes, gills, adductor muscles, mantles, hearts, and digestive glands. Each tissue was dissected from three oysters. Developmental stage expression profiles of *PfSMAD4* were analyzed in fertilized eggs, 2–4 cell stage, blastocysts, the trochophore, D-shaped larvae, umbo larvae, eye-spot larvae, spats and juveniles. *β-actin* was expressed stably in all tested tissues and developmental stages. Three repetitions of the reaction were performed.

### Subcellular localization

Subcellular localization of PfSMAD4 was performed by immunofluorescence assays. The HEK293T cells were seeded onto cover slips (10 mm × 10 mm) in a 12-well plate. After transfection for 48 h, the HEK293T cells were fixed with 4 % paraformaldehyde and then the coverslips were blocked using 2 % bovine serum albumin at room temperature for 30 min. Cells were incubated either with anti-myc antibody (1:60) or preimmune mouse serum (1:60) for 1 h, rinsed with PBS three times for 10 min and then incubated with FITC-conjugated goat anti-mouse antibodies (Pierce, Rockford, IL, USA) for a further hour. Finally, cells were stained with DAPI (1 mg/ml) and observed under fluorescence microscopy.

### RNAi experiments

RNA interference (RNAi) was performed as described in Suzuki et al. [48], with some modifications. The primers used for generating *PfBMP2* and *PfSMAD4* dsRNA were RBMP2F:**GCGTAATACGACTCACTATAGGGAGA**CATCCCGCAGTATTAAAGTGG, RBMP2R:**GCGTAATACGACTCACTATAGGGAGA**CCGACATCCGCATCCTTCAAC; RSMAD4F:**GCGTAATACGACTCACTATAGGGAGA**TTATGCCAGGATTTGGAGAT; RSMAD4R:**GCGTAATACGACTCACTATAGGGAGA**GAGGCTTGAGACTGAGGAG. The T7 promoter sequence is bold. For GFP, pEGFP-C1 (Clontech) was used as the template. A RiboMAX Large Scale RNA Production System (T7) kit (Promega) was used to synthesize and purify the dsRNA. RNase-free DNase I (TaKaRa, Otsu, Japan) was used to digest the template DNA. The *PfBMP2* dsRNA and *PfSMAD4* dsRNA were diluted to 80 μg/100 μl using PBS, and 100 μl solutions were injected into pearl oyster adductors. PBS and dsRNA-GFP were used as controls. Total RNA from the mantle tissue of each oyster was extracted 7 days after injection and used to synthesize the first strand cDNA as described above. qPCR was used to quantify the expression levels of *PfBMP2* and *PfSMAD4*, where *β-actin* was used as an internal reference. The qPCR primers that were designed for *PfSMAD4* and *β-actin* were the same sequences as in the distribution experiments above. The shell of each oyster was thoroughly washed with Milli-Q water and air-dried. It was then cut into pieces and mounted on the scanner with the inner nacreous surface face-up, sputter-coated with 10 nm-thick gold, and analyzed using scanning electron microscopy (SEM, S-3400 N, Hitachi, Tokyo, Japan).

### Luciferase assays

HEK293T cells (1.5 × 10^5^ cells/well) were seeded onto 48-well plates. Cells were transfected with the pGL3 reporter gene in the absence or presence of *PfBMP2* expression vectors. The total amount of DNA (1.0 μg) was kept constant with empty vectors. For normalization of transfection efficiencies, 0.1 μg of *Renilla* (sea pansy) luciferase expression plasmid (pRL-TK, Promega) was included in the transfection experiments. Transfected cells were lysed and subjected to luciferase assays using luciferin substrate (Promega) following the manufacturer's instructions. The assays were performed in triplicates.

### Statistical analysis

Data were analyzed by one-way analysis of variance (ANOVA) with default parameters or the Student’s t-test to identify differences between groups. Differences were considered statistically significant when *P* values were lower than 0.05.

## Abbreviations

BMP: bone morphogenic proteins
DAPI: 6-diamidino-2-pheny- lindole
DMEM: dulbecco's modified eagle media
dsRNA: double-stranded RNA
FBS: fetal bovine serum
FITC: fluorescein isothiocyanate
GFP: green fluorescent protein
PBS: phosphate-buffered Saline
qPCR: quantitative PCR
RNAi: RNA interference
SEM: scanning electron microscope
SMAD: mothers against DPP homologs
TGF: transforming growth factor

## References

[1] Evans A, Suo Z, Wang R, Aksay I, He M, Hutchinson J (2001). Model for the robust mechanical behavior of nacre. J Mater Res.

[2] Wang R, Suo Z, Evans A, Yao N, Aksay I (2001). Deformation mechanisms in nacre. J Mater Res.

[3] Zentz F, Bédouet L, Almeida MJ, Milet C, Lopez E, Giraud M (2001). Characterization and quantification of chitosan extracted from nacre of the abalone Haliotis tuberculata and the oyster Pinctada maxima. Mar Biotechnol.

[4] Wada K (1972). Nucleation and growth of aragonite crystals in the nacre of some bivalve molluscs. Biomineralization.

[5] Addadi L, Weiner S (1997). Biomineralization: A pavement of pearl. Nature.

[6] Attisano L, Wrana JL (2002). Signal transduction by the TGF-β superfamily. Science.

[7] Xiao Y-T, Xiang L-X, Shao J-Z (2007). Bone morphogenetic protein. Biochem Biophys Res Commun.

[8] Canalis E, Economides AN, Gazzerro E (2003). Bone morphogenetic proteins, their antagonists, and the skeleton. Endocr Rev.

[9] Cho Y-D, Yoon W-J, Woo K-M, Baek J-H, Park J-C, Ryoo H-M (2010). The canonical BMP signaling pathway plays a crucial part in stimulation of dentin sialophosphoprotein expression by BMP-2. J Biol Chem.

[10] Cao X, Chen D (2005). The BMP signaling and in vivo bone formation. Gene.

[11] Retting KN, Song B, Yoon BS, Lyons KM (2009). BMP canonical Smad signaling through Smad1 and Smad5 is required for endochondral bone formation. Development.

[12] Padgett RW, Wozney JM, Gelbart WM (1993). Human BMP sequences can confer normal dorsal-ventral patterning in the Drosophila embryo. Proc Natl Acad Sci.

[13] Zhang Y, Musci T, Derynck R (1997). The tumor suppressor Smad4/DPC 4 as a central mediator of Smad function. Curr Biol.

[14] Das P, Maduzia LL, Wang H, Finelli AL, Cho S-H, Smith MM, Padgett RW (1998). The Drosophila gene Medea demonstrates the requirement for different classes of Smads in dpp signaling. Development.

[15] Nohe A, Keating E, Knaus P, Petersen NO (2004). Signal transduction of bone morphogenetic protein receptors. Cell Signal.

[16] Shi Y, Massagué J (2003). Mechanisms of TGF-β signaling from cell membrane to the nucleus. Cell.

[17] Heldin C-H, Miyazono K, Ten Dijke P (1997). TGF-β signalling from cell membrane to nucleus through SMAD proteins. Nature.

[18] Attisano L, Lee-Hoeflich ST. The smads. Genome Biol. 2001; 2(8): REVIEWS3010. Epub 2001 Aug 2 10.1186/gb-2001-2-8-reviews3010 PMC138956 11532220

[19] Shioda T, Lechleider RJ, Dunwoodie SL, Li H, Yahata T, De Caestecker MP, Fenner MH, Roberts AB, Isselbacher KJ (1998). Transcriptional activating activity of Smad4: roles of SMAD hetero-oligomerization and enhancement by an associating transactivator. Proc Natl Acad Sci U S A.

[20] Kin K, Kakoi S, Wada H (2009). A novel role for *dpp* in the shaping of bivalve shells revealed in a conserved molluscan developmental program. Dev Biol.

[21] Shimizu K, Sarashina I, Kagi H, Endo K (2011). Possible functions of Dpp in gastropod shell formation and shell coiling. Dev Genes Evol.

[22] Nederbragt AJ, van Loon AE, Dictus WJ (2002). Expression of *Patella vulgata* Orthologs of *engrailed* and *dpp-BMP2/4* in Adjacent Domains during Molluscan Shell Development Suggests a Conserved Compartment Boundary Mechanism. Dev Biol.

[23] Shimizu K, Iijima M, Setiamarga DH, Sarashina I, Kudoh T, Asami T, Gittenberger E, Endo K (2013). Left-right asymmetric expression of dpp in the mantle of gastropods correlates with asymmetric shell coiling. EvoDevo.

[24] Hashimoto N, Kurita Y, Wada H (2012). Developmental role of *dpp* in the gastropod shell plate and co-option of the *dpp* signaling pathway in the evolution of the operculum. Dev Biol.

[25] Gong N, Shangguan J, Liu X, Yan Z, Ma Z, Xie L, Zhang R (2008). Immunolocalization of matrix proteins in nacre lamellae and their *in vivo* effects on aragonitic tablet growth. J Struct Biol.

[26] Miyashita T, Hanashita T, Toriyama M, Takagi R, Akashika T, Higashikubo N (2008). Gene cloning and biochemical characterization of the BMP-2 of Pinctada fucata. Biosci Biotechnol Biochem.

[27] Miyashita T. Studies on the Pinctada fucata BMP-2 Gene: Structural Similarity and Functional Conservation of Its Osteogenic Potential within the Animal Kingdom. International Journal of Zoology. 2013;2013.

[28] Luo Y-J, Takeuchi T, Koyanagi R, Yamada L, Kanda M, Khalturina M, Fujie M, Yamasaki S-I, Endo K, Satoh N (2015). The Lingula genome provides insights into brachiopod evolution and the origin of phosphate biomineralization. Nat Commun.

[29] Liu G, Huan P, Liu B (2014). Cloning and expression patterns of two Smad genes during embryonic development and shell formation of the Pacific oyster *Crassostrea gigas*. Chinese J Oceanol Limnol.

[30] Pierreux CE, Nicolás FJ, Hill CS (2000). Transforming growth factor β-independent shuttling of Smad4 between the cytoplasm and nucleus. Mol Cell Biol.

[31] Inman GJ, Nicolás FJ, Hill CS (2002). Nucleocytoplasmic shuttling of Smads 2, 3, and 4 permits sensing of TGF-β receptor activity. Mol Cell.

[32] Zaidi SK, Sullivan AJ, Van Wijnen AJ, Stein JL, Stein GS, Lian JB (2002). Integration of Runx and Smad regulatory signals at transcriptionally active subnuclear sites. Proc Natl Acad Sci U S A.

[33] Chen G, Deng C, Li Y-P (2012). TGF-β and BMP signaling in osteoblast differentiation and bone formation. Int J Biol Sci.

[34] Tan X, Weng T, Zhang J, Wang J, Li W, Wan H, Lan Y, Cheng X, Hou N, Liu H (2007). Smad4 is required for maintaining normal murine postnatal bone homeostasis. J Cell Sci.

[35] Le Goff C, Mahaut C, Abhyankar A, Le Goff W, Serre V, Afenjar A, Destrée A, di Rocco M, Héron D, Jacquemont S (2012). Mutations at a single codon in Mad homology 2 domain of SMAD4 cause Myhre syndrome. Nat Genet.

[36] Lazzereschi D, Nardi F, Turco A, Ottini L, D'Amico C, Mariani-Costantini R, Gulino A, Coppa A (2005). A complex pattern of mutations and abnormal splicing of Smad4 is present in thyroid tumours. Oncogene.

[37] Maru D, Wu T-T, Canada A, Houlihan PS, Hamilton SR, Rashid A (2004). Loss of chromosome 18q and DPC4 (Smad4) mutations in appendiceal adenocarcinomas. Oncogene.

[38] Hohenstein P, Molenaar L, Elsinga J, Morreau H, van der Klift H, Struijk A, Jagmohan‐Changur S, Smits R, van Kranen H, van Ommen GJB (2003). Serrated adenomas and mixed polyposis caused by a splice acceptor deletion in the mouse Smad4 gene. Genes Chromosomes Cancer.

[39] Warmflash A, Zhang Q, Sorre B, Vonica A, Siggia ED, Brivanlou AH (2012). Dynamics of TGF-β signaling reveal adaptive and pulsatile behaviors reflected in the nuclear localization of transcription factor Smad4. Proc Natl Acad Sci U S A.

[40] Tao S, Sampath K (2010). Alternative splicing of SMADs in differentiation and tissue homeostasis. Dev Growth Differ.

[41] Westbroek P, Marin F (1998). A marriage of bone and nacre. Nature.

[42] De Robertis E (2008). Evo-devo: variations on ancestral themes. Cell.

[43] Samuel G, Miller D, Saint R (2001). Conservation of a DPP/BMP signaling pathway in the nonbilateral cnidarian Acropora millepora. Evol Dev.

[44] Eivers E, Demagny H, De Robertis E (2009). Integration of BMP and Wnt signaling via vertebrate Smad1/5/8 and *Drosophila* Mad. Cytokine Growth F R.

[45] Kobayashi A, Sasakura Y, Ogasawara M, Makabe KW (1999). A maternal RNA encoding smad1/5 is segregated to animal blastomeres during ascidian development. Dev Growth Differ.

[46] Yu X, Li J, Liu H, Li X, Chen S, Zhang H, Xu A (2011). Identification and expression of amphioxus AmphiSmad1/5/8 and AmphiSmad4. Sci China Life Sci.

[47] Shrey K, Suchit A, Nishant M, Vibha R (2009). RNA interference: emerging diagnostics and therapeutics tool. Biochem Biophys Res Commun.

[48] Suzuki M, Saruwatari K, Kogure T, Yamamoto Y, Nishimura T, Kato T, Nagasawa H (2009). An acidic matrix protein, Pif, is a key macromolecule for nacre formation. Science.

[49] Fang D, Xu G, Hu Y, Pan C, Xie L, Zhang R (2011). Identification of genes directly involved in shell formation and their functions in pearl oyster, Pinctada fucata. PLoS ONE.

[50] Jiao Y, Wang H, Du X, Zhao X, Wang Q, Huang R, Deng Y (2012). Dermatopontin, a shell matrix protein gene from pearl oyster *Pinctada martensii*, participates in nacre formation. Biochem Biophys Res Commun.

[51] Funabara D, Ohmori F, Kinoshita S, Koyama H, Mizutani S, Ota A, Osakabe Y, Nagai K, Maeyama K, Okamoto K (2014). Novel Genes Participating in the Formation of Prismatic and Nacreous Layers in the Pearl Oyster as Revealed by Their Tissue Distribution and RNA Interference Knockdown. PLoS ONE.

[52] Larkin M, Blackshields G, Brown N, Chenna R, McGettigan PA, McWilliam H, Valentin F, Wallace IM, Wilm A, Lopez R (2007). Clustal W and Clustal X version 2.0. Bioinformatics.

[53] Tamura K, Peterson D, Peterson N, Stecher G, Nei M, Kumar S (2011). MEGA5: molecular evolutionary genetics analysis using maximum likelihood, evolutionary distance, and maximum parsimony methods. Mol Biol Evol.

